# Low skeletal muscle mass assessed directly from the 3rd cervical vertebra can predict pharyngocutaneous fistula risk after total laryngectomy in the male population

**DOI:** 10.1007/s00405-021-07127-3

**Published:** 2021-10-19

**Authors:** Maria Casasayas, Jacinto García-Lorenzo, Beatriz Gómez-Ansón, Victoria Medina, Alejandro Fernández, Miquel Quer, Xavier León

**Affiliations:** 1 Servicio de Otorrinolaringología, Hospital de la Santa Creu i Sant Pau, Universitat Autònoma de Barcelona, C/Mas Casanovas, 90, 08041 Barcelona, Spain; 2 Servicio de Otorrinolaringología, Hospital del Mar, Barcelona, Spain; 3 Servicio de Radiodiagnóstico, Unidad de Neuro-Radiología, Hospital de la Santa Creu i Sant Pau, Universitat Autònoma de Barcelona, Barcelona, Spain; 4 Servicio de Medicina Nuclear, Hospital de la Santa Creu i Sant Pau, Universitat Autònoma de Barcelona, Barcelona, Spain; 5 Centro de Investigación Biomédica en Red de Bioingeniería, Biomateriales y Nanomedicina (CIBER-BBN), Madrid, Spain

**Keywords:** Head and neck squamous cell carcinoma, Pharyngocutaneous fistula, Total laryngectomy, Sarcopenia, Computed tomography

## Abstract

**Purpose:**

Skeletal muscle mass (SMM) loss and sarcopenia have been identified as risk factors for postoperative complications. The aim of this study was to investigate the relationship between pharyngocutaneous fistula (PCF) formation after total laryngectomy (TL) and SMM assessed from a computed tomography image of the 3rd cervical vertebra (C3).

**Methods:**

Retrospective study of 86 male patients who underwent TL between 2013 and 2019 in a single institution. We excluded women from the analysis due to our limited sample. SMM was determined from cross-sectional muscle area (CSMA) measurement at C3 using the ImageJ software. Results were compared with those for the skeletal muscle mass index (SMMI) calculated from the estimated measure at 3rd lumbar vertebra (L3).

**Results:**

PCF formation occurred in 21/86 patients. According to the CSMA at a C3 cut-off of 35.5cm2, of 18 patients (20.9%) with low SMM, 9 developed PCFs (50.0%). Among patients with normal SMM (*n* = 68, 79.1%), 12 developed PCFs (17.6%). The CSMA at C3 was the only variable significantly associated with PCF risk, which was 4.7 times greater in patients with low SMM (*p* = 0.007). Sarcopenia was more frequent in underweight patients (*p* = 0.0001), patients undergoing extended surgeries (*p* = 0.003), or presenting preoperative anaemia (*p* = 0.009) or hypoalbuminemia (*p* = 0.027).

**Conclusion:**

Measuring the CSMA at C3 obtained results equivalent to those obtained by calculating the SMMI at L3, suggesting that direct SMM assessment from C3 is a useful approach to evaluating PCF formation risk after TL.

**Supplementary Information:**

The online version contains supplementary material available at 10.1007/s00405-021-07127-3.

## Introduction

For some advanced laryngeal and hypopharyngeal tumours, total laryngectomy (TL) is the initial treatment or a salvage treatment after local recurrence. A postoperative complication of TL is the formation of a pharyngocutaneous fistula (PCF). While several observational studies and meta-analyses review predisposing factors for PCF formation [1–5], there is currently no consensus as to which of those factors is the most predictive. In a study performed in our hospital [6], factors identified as being significantly related to PCF formation were the extent of surgery and the presence of postoperative haemoglobin levels below 99 g/L.

A growing concern regarding the nutritional status of patients with cancer highlights sarcopenia as a risk factor for treatment-associated complications [7–12]. Sarcopenia, defined by the European Working Group on Sarcopenia in Older People (EWGSOP) [13] as progressive and generalized skeletal muscle impairment, is diagnosed by objectifying decreased muscle strength and altered quantity or quality of muscle tissue. Patients with head and neck carcinomas (HNSCC) frequently lose muscle mass due to the metabolic characteristics of their tumour, to its location at the level of the upper airway and digestive tract, and to important sequelae or toxicities produced by treatments [14]. The reported prevalence of sarcopenia in patients with HNSCC varies widely, from as low as 6% [15] to as high as 77% [9].

Of several techniques described to evaluate sarcopenia, cervical computed tomography (CT), a component of routine tumour staging studies, has been proposed as especially useful for patients with HNSCC [16]. From CT images obtained at the level of the 3rd cervical vertebra (C3), muscle tissue can be accurately quantified so as to determine body composition. Since Swartz et al. [16] demonstrated that skeletal muscle mass (SMM) measured at C3 is proportional to muscle mass measured at the 3rd lumbar vertebra (L3)—the standard location for quantifying SMM—that formula has been used in most studies of sarcopenia in patients with HNSCC to estimate the cross-sectional muscle area (CSMA) at L3 from the area measured at C3 [7, 10, 11, 17–20]. A skeletal muscle mass index (SMMI) is then obtained by dividing the L3 area by the squared height of the patient.

However, use of the Swartz et al. [16] formula is complicated in routine clinical practice. According to the reviewed literature, only Jung et al. [21] have evaluated diminished SMM based on measurements at C3; in that study, which included 305 patients with advanced HNSCC in different locations, grouped according to treatment (surgical vs radiotherapy ± chemotherapy), survival was analysed according to the CSMA at the C3 level, with the authors observing that patients with a CSMA at C3 of less than 56.3 cm^2^ had poorer overall survival.

There is no consensus on what cut-off point should be used to define low SMM, but it is probably necessary to set a specific cut-off point for each sex due to differences in muscle constitution. Prado et al. [22], whose sex-specific cut-offs for defining sarcopenia have been used multiple times in the literature [9, 23–25], found the mean CSMA at L3 value to be higher in obese men than in obese women (180.2 cm^2^ and 125.1 cm^2^, respectively). In our institution, our female population undertaking a TL is limited; therefore, we decided to exclude women from this study.

The aim of the present study was to analyse the relationship between low SMM, as determined from the CSMA measured directly from a CT scan slice of C3, and postoperative PCF formation in male patients treated with TL.

## Materials and methods

The study followed the ethical standards of the latest version of the Helsinki Declaration, and was evaluated and authorized by our hospital’s Clinical Research Ethics Committee [reference 19/126 (OBS)]. Given the retrospective nature of the study, the informed consent of the patients included in the study was not considered necessary.

### Patient description

We retrospectively reviewed all TL procedures performed consecutively between 2013 and 2019 in our hospital. For 105 of the 113 patients who underwent TL, preoperative cervical CT images were available that allowed an evaluation of the SMM. We only included patients with an available imaging test 2 months prior to the surgery (median 34.5 days, range 2–61 days); therefore, 6 patients were excluded. We excluded 13 women who underwent a TL during the study period. In total, 86 male patients were included, most of them treated with simple TL (*n* = 67), extended TL with pharyngectomy without reconstruction (*n* = 11), and extended TL with pharyngectomy and reconstruction with a pectoralis major flap (*n* = 8). All eight patients undergoing flap reconstruction had been implanted with a salivary bypass tube.

Surgery was the initial treatment in 43 cases (50.0%), and was salvage treatment in 31 cases (36.0%): 13 (41.9%) after radiotherapy; 2 (6.5%) after surgery and radiotherapy; 13 (41.9%) after chemo-radiotherapy; and 3 (9.7%) after bioradiotherapy. In the remaining 12 cases (14.0%), the surgical indication was resection of a second tumour located in the larynx or hypopharynx of patients previously treated with radiotherapy. Of the 86 included patients, 43 (50.0%) had received radiotherapy prior to surgery, and in 74 patients (86.1%), surgical treatment included neck dissection, unilaterally in 12 cases (14.0%) and bilaterally in 62 cases (72.1%).

All patients received antibiotic prophylaxis, usually with amoxicillin–clavulanic acid: 2 g administered 30 min before surgery, and 1 g administered every 4 h during surgery. Patients allergic to penicillin were administered the combination clindamycin 900 mg + gentamicin 5 mg/kg. Enteral nutrition by nasogastric tube was initiated on the 1st postoperative day. If no wound complications occurred, oral intake was started on the 7th postoperative day in non-irradiated patients, and on the 12th postoperative day in patients who had previously received radiotherapy or undergone pectoralis major flap reconstruction.

### Data analysis

A fistula was defined as any clinical suspicion of salivary leak confirmed by oral administration of methylene blue dye. When a PCF was confirmed, the initial treatment was conservative management involving antibiotic therapy, continuation of enteral nutrition, and daily local wound care. In cases that did not respond adequately to conservative management, pectoralis major flap reconstruction was considered on a case-by-case basis [26].

The following patient-related variables were assessed: sex; age; alcohol use; tobacco use; diabetes mellitus; and American Society of Anesthesiologists (ASA) physical status. Patients were classified into 1 of 3 categories according to their use of alcohol and tobacco, as follows: no use; moderate use (< 20 cigarettes/day and/or < 80 g alcohol/day); or heavy use (≥ 20 cigarettes/day or ≥ 80 g alcohol/day). The body mass index (BMI) was calculated for each patient, with patients grouped according to the World Health Organization (WHO) classification [27] as follows: underweight (< 18.5 kg/m^2^); normo-overweight (18.5–29.9 kg/m^2^); or obese (> 30 kg/m^2^). Also evaluated were the following variables: tumour extension and location; previous radiotherapy or chemo-radiotherapy; surgery type; automatic suture use; neck dissection; voice prosthesis placement; preoperative and postoperative haemoglobin levels (according to WHO [28] anaemia criteria); and preoperative albumin and protein levels. Table 1 summarizes the patient characteristics.

**Table 1:** Patient descriptive characteristics

Characteristic		*n* (%)/mean (SD)
Age (years)		65.7 (10.3)
Tobacco/alcohol use	None	1 (1.2%)
	Moderate	14 (16.3%)
	Heavy	71 (82.6%)
ASA physical status	I–II	41 (47.7%)
	III–IV	45 (52.3%)
Diabetes mellitus	No	62 (72.1%)
	Yes	24 (27.9%)
Body mass index (kg/m^2^)		25.4 (4.7)
Subsite location	Supraglottis	24 (27.9%)
	Glottis	51 (59.3%)
	Hypopharynx	11 (12.8%)
Tumour stage (pT)	pT2	12 (14.0%)
	pT3	19 (22.1%)
	pT4	55 (64.0%)
Previous radiotherapy	No	43 (50.0%)
	Yes	43 (50.0%)
Surgery type	Simple TL	67 (77.9%)
	Extended TL ± reconstruction	19 (22.1%)
Neck dissection	No	12 (14.0%)
	Unilateral	12 (14.0%)
	Bilateral	62 (72.1%)
Voice prosthesis	No	76 (88.4%)
	Yes	10 (11.6%)
Automatic suture	No	56 (65.1%)
	Yes	30 (34.9%)
Preoperative haemoglobin (g/L)		140.2 (19.4)
Postoperative haemoglobin (g/L)		109.4 (15.4)
Preoperative protein (g/L)^†^		64.9 (9.4)
Preoperative albumin (g/L)^‡^		38.6 (5.3)

*ASA* American Society of Anesthesiologists, *TL* total laryngectomy

^†^Data available from 44 patients

^‡^Data available from 46 patients

### CSMA measurements

All images, in Digital Imaging and Communications in Medicine (DICOM) format, were anonymized by a person not involved in the study, for analysis by a single researcher (MC), who had previously received training guided by the Radiodiagnosis Department.

The CSMA was measured from CT or positron emission tomography (PET-CT) images obtained in the 3 months prior to surgery. Images were analysed using Image J (software v1.44p) [29] and Fiji accessories [30]. The axial slice corresponding to C3 was selected following Swartz et al.’s [16] guidelines, i.e., axial C3 slices were scrolled through in the caudo-cranial direction and selected was the first slice that fully showed the vertebral arch, the transverse processes, and the spinous process. The CSMA was evaluated for the prevertebral and nuchal musculature and sternocleidomastoid muscles. In patients with pathological lymph nodes infiltrating part of the musculature to be evaluated, a theoretical line was drawn to delimit the lymph node, and possible infiltrated muscle tissue was excluded as non-viable. The CSMA was defined as the sum of the areas of each muscle bundle (right and left sternocleidomastoid and perivertebral musculature) at the C3 level.

Intraobserver and interobserver validation studies of 10 CT images were based on a repeat analysis and an analysis by a different investigator (CR), respectively. Correlation coefficients were 0.95 or greater for measurements at the C3 level. Figure S1 (supplementary material) shows the correlations obtained in the validation studies.

### Statistical analysis

SPSS version 17.0 was used for data analysis. Results were considered to be statistically significant at *p* < 0.05.

A recursive partitioning analysis (RPA) using the classification and regression tree (CRT) method was used to obtain a cut-off point to categorize the continuous CSMA variable (independent variable) according to PCF formation (dependent variable). Patients with CSMA values below the cut-off obtained with the RPA were considered sarcopenic.

A univariate analysis of PCF formation was performed for each evaluated variable, including the CSMA categories obtained in the RPA, using either the Chi-square test or Fisher’s exact test depending on the application conditions. A multivariate analysis was performed with a logistic regression model, considering PCF as the dependent variable, and the remaining variables as independent variables. We excluded of the multivariate analysis the preoperative albumin and protein levels due to a lack of data for 40 and 42 patients, respectively. The patient who made no use of alcohol or tobacco (*n* = 1) was included in the moderate alcohol/tobacco use group.

To validate our results, the study was repeated, this time measuring the CSMA at L3 using the Swartz et al. [16] formula and calculating the SMMI.

CSMA values were analysed according to the different clinical variables using the Student’s t test or the analysis of variance (ANOVA) test. Correlation between continuous variables was evaluated using Pearson’s correlation coefficient.

## Results

### PCF and sarcopenia

A total of 21 patients (24.4%) developed PCFs after TL. Considering PCF formation as the dependent variable, the RPA cut-off point for the CSMA at C3 was 35.5 cm^2^. According to this threshold value, 18 patients (20.9%) presented low SMM, and of these, 9 patients (50.0%) developed PCFs; of the 68 patients with a normal SMM (79.1%), 12 (17.6%) developed PCFs. The risk of PCF formation was thus 4.67 times greater in low SMM patients than in patients with normal SMM (95% CI 1.53–14.23; *p* = 0.007).

Table 2 shows the percentage of patients with PCF formation according to the different analysed variables. Apart from the CSMA at C3, tumour location, surgery type, and suture type proved to be significantly related to PCF formation. Thus, PCF frequency was greater for hypopharyngeal tumours (63.6%) than for supraglottic tumours (20.8%) or glottic tumours (17.6%) (*p* = 0.008); for extended TL with pharyngectomy (52.6%) than for simple TL (16.4%) (*p* = 0.002); and for manual suture (32.1%) than for automatic suture (10.0%) (*p* = 0.023).

**Table 2:** Univariate analysis of patients with pharyngocutaneous fistula

Characteristic		% PCF	*p* value
Age	≤ 65 years	23.8	0.898
	> 65 years	25.0	
Tobacco/alcohol use	Moderate	33.3	0.508
	Heavy	22.5	
ASA physical status	I–II	24.4	0.995
	III–IV	24.4	
Diabetes mellitus	No	24.2	0.938
	Yes	25.0	
CSMA at C3 (low SMM = ≤ 35.5 cm^2^)	≤ 35.5 cm^2^	50.0	0.011*
	> 35.5 cm^2^	17.6	
Body mass index	Underweight (< 18.5 kg/m^2^)	37.5	0.649
	Normo-overweight (18.5–29.9 kg/m^2^)	23.8	
	Obese (> 30 kg/m^2^)	20.0	
Subsite location	Supraglottis	20.8	0.008*
	Glottis	17.6	
	Hypopharynx	63.6	
Tumour stage (pT)	pT2	25.0	0.621
	pT3	15.8	
	pT4	27.3	
Previous radiotherapy	No	25.6	0.802
	Yes	23.3	
Surgery type	Simple TL	16.4	0.002*
	Extended TL ± reconstruction	52.6	
Automatic suture	No	32.1	0.023*
	Yes	10.0	
Neck dissection	No	25.0	1.000
	Unilateral	25.0	
	Bilateral	24.2	
Voice prosthesis	No	26.3	0.439
	Yes	10.0	
Preoperative protein^†^	≤ 60 g/L	23.1	1.000
	> 60 g/L	19.4	
Preoperative albumin^‡^	≤ 35 g/L	21.4	1.000
	> 35 g/L	18.8	
Preoperative anaemia	No	23.3	0.722
	Yes	26.9	
Postoperative anaemia	No	14.3	1.000
	Yes	25.3	

*ASA* American Society of Anesthesiologists, *C3* 3rd cervical vertebra, *CSMA* cross-sectional muscle area, *PCF* pharyngocutaneous fistula, *SMM* skeletal muscle mass, *TL* total laryngectomy

*Statistical significance *p* < 0.05

^†^Data available from 44 patients

^‡^Data available from 46 patients

Table 3 shows the results of the multivariate study, considering PCF formation as the dependent variable and the remaining variables as independent variables. The results show that the CSMA at C3 was the only variable that was significantly associated with PCF risk: the hazard ratio (HR) for PCF formation in patients with a CSMA at C3 of ≤ 35.5 cm2 was 9.88 times greater than for patients with a CSMA at C3 of > 35.5 cm^2^ (95% CI: 1.40–69.73; *p* = 0.022).

**Table 3:** Pharyngocutaneous fistula risk: multivariate analysis including cross-sectional muscle area at the 3rd cervical vertebra

Characteristic		PCF HR (CI 95%)	*p* value
Age	(Continuous variable)	0.97 (0.90–1.05)	0.488
Tobacco/alcohol use	Moderate	1	0.051
	Heavy	0.13 (0.02–1.01)	
ASA physical status	I-II	1	0.633
	III-IV	0.69 (0.15–3.14)	
Diabetes mellitus	No	1	0.144
	Yes	3.27 (0.67–15.96)	
Body mass index (kg/m^2^)	(Continuous variable)	1.12 (0.94–1.33)	0.202
CSMA at C3 (low SMM = ≤ 35.5 cm^2^)	> 35.5 cm^2^	1	0.022*
	≤ 35.5 cm^2^	9.88 (1.40–69.73)	
Subsite location	Supraglottis	1	
	Glottis	2.62 (0.38–17.90)	0.326
	Hypopharynx	6.41 (0.49–83.85)	0.156
Tumour stage (pT)	pT2	1	
	pT3	1.24 (0.12–13.08)	0.857
	pT4	1.13 (0.14–8.88)	0.911
Previous radiotherapy	No	1	0.823
	Yes	0.84 (0.17–4.04)	
Surgery type	Simple TL	1	0.171
	Extended TL ± reconstruction	5.75 (0.47–70.22)	
Automatic suture	No	1	0.354
	Yes	0.42 (0.06–2.64)	
Neck dissection	No	1	
	Unilateral	0.30 (0.02–4.34)	0.375
	Bilateral	0.70 (0.08–5.78)	0.739
Voice prosthesis	No	1	0.415
	Yes	0.33 (0.02–4.77)	
Preoperative anaemia	No	1	0.523
	Yes	0.58 (0.11–3.08)	
Postoperative anaemia	No	1	0.510
	Yes	2.29 (0.19–27.02)	

*ASA* American Society of Anesthesiologists, *C3* 3rd cervical vertebra, *CSMA* cross-sectional muscle area, *HR* hazard ratio, *PCF* pharyngocutaneous fistula, *SMM* skeletal muscle mass, *TL* total laryngectomy

*Statistical significance *p* < 0.05

The study was repeated using the standard Swartz et al. [16] approach to measuring sarcopenia, based on C3-to-L3 conversion of the CSMA and calculating the SMMI by dividing the obtained value by the squared patient height. The RPA cut-off point based on the SMMI value and PCF formation was 47.7 cm^2^/m^2^; accordingly, 46.5% (n = 40) of patients had decreased SMM, and of these, 37.5% (*n* = 15) developed PCFs, compared to 13.0% of 46 patients with normal SMM. PCF risk was 4.00 times greater in patients with low SMM than in patients with normal SMM (95% CI 1.37–11.67; *p* = 0.011). Table S1 (supplementary material) shows the multivariate analysis results, with the evaluation of SMM according to the SMMI included as an independent variable; as was the case for the CSMA at C3, the only variable that was significantly associated with PCF risk was low SMM. This risk was 8.5 times greater for patients with an SMMI of ≤ 47.7 cm^2^/m^2^ than for patients with an SMMI of > 47.7 cm^2^/m^2^ (95% CI 1.22–59.36; *p* = 0.031).

### Patients with low SMM

Table 4 shows the proportion of patients with low SMM measured according to the CSMA at C3 for each studied variable. Table S2 (supplementary material) shows values for the CSMA at C3 expressed as the mean (SD) for each variable.

**Table 4:** Percentage of patients with low skeletal muscle mass measured directly at the 3rd cervical vertebra according to different variables

Characteristic		*n*	Low SMM (%)	*p* value
Age (years)	≤ 65	42	19.0	0.675
	> 65	44	22.7	
Tobacco/alcohol use	Moderate	15	6.7	0.177
	Heavy	71	23.9	
ASA physical status	I–II	41	12.2	0.057
	III-IV	45	28.9	
Diabetes mellitus	No	62	24.2	0.232
	Yes	24	12.5	
Body mass index (kg/m^2^)	Underweight (< 18.5 kg/m^2^)	8	87.5	0.0001*
	Normo-overweight (18.5–29.9 kg/m^2^)	63	17.5	
	Obesity (> 30 kg/m^2^)	15	0.0	
Subsite location	Supraglottis	24	25.0	0.052
	Glottis	51	13.7	
	Hypopharynx	11	45.5	
Tumour stage (pT)	pT2	12	8.3	0.399
	pT3	19	15.8	
	pT4	55	25.5	
Previous radiotherapy	No	43	27.9	0.112
	Yes	43	14.0	
Surgery type	Simple TL	67	13.4	0.003*
	Extended TL ± reconstruction	19	47.4	
Automatic suture	No	56	30.4	0.003*
	Yes	30	3.3	
Neck dissection	No	12	16.7	0.917
	Unilateral	12	25.0	
	Bilateral	62	21.0	
Voice prosthesis	No	76	22.4	0.681
	Yes	10	10.0	
Preoperative protein^†^	≤ 60 g/L	13	15.4	0.697
	> 60 g/L	31	25.8	
Preoperative albumin^‡^	≤ 35 g/L	14	50.0	0.027*
	> 35 g/L	32	15.6	
Preoperative anaemia	No	60	13.3	0.009*
	Yes	26	38.5	
Postoperative anaemia	No	7	14.3	1.000
	Yes	79	21.5	

*ASA* American Society of Anesthesiologists, *SMM* skeletal muscle mass, *TL* total laryngectomy

*Statistical significance *p* < 0.05

^†^Data available from 44 patients

^‡^Data available from 46 patients

Differences were observed according to BMI, surgery extension, the use of automatic suture, and preoperative anaemia and hypoalbuminemia. As for BMI, the higher the value, the greater the observed CSMA at C3 (*p* = 0.0001); based on the WHO BMI classification, 87.5% of underweight, 17.5% of normo-overweight, and 0.0% of obese patients were observed to have low SMM (*p* = 0.0001). Patients with extended surgeries suffered more SMM depletion than patients with simple TL (47.4% versus 13.4%, *p* = 0.003). In the group of patients in which an automatic suture was used, the prevalence of SMM depletion was lower than in patients sutured manually (3.3% versus 30.4%).

Also, patients with preoperative anaemia presented more risk of low SMM, observed in 38.5% of those patients compared to 13.3% for patients without anaemia (*p* = 0.009). Even though the available data regarding preoperative albumin levels were limited, 50.0% of patients with values below 35 g/L presented low SMM, while for patients with normal values, it was of 15.6% (*p* = 0.027). No differences were observed regarding patients with low SMM as a function of previous radiotherapy treatment (*p* = 0.112).

## Discussion

### Low SMM prevalence

In our study of the relationship between low SMM and PCF formation after treatment with TL in male patients, SMM was evaluated considering the CSMA calculated from CT slices obtained at the C3 level. We found, on the basis of direct measurement of the CSMA at C3 and a cut-off point of 35.5 cm^2^, that 20.9% of our patients presented low SMM.

In preliminary phases of this study, we observed that the 13 women in our cohort had lower mean CSMA at C3 values than men (30.22 cm^2^ vs 41.02 cm^2^; *p* = 0.0001) (Figure S2—supplementary material). Given the small number of female patients, it was not possible for us to establish a specific cut-off for women, so we excluded those patients from the study.

Calculating a sex-specific cut-off for men allowed a more restrictive cut-off point to be obtained, which probably is more appropriate for the identification of patients at a higher PCF risk, as, from the perspective of—possibly costly and individualized—prevention, it would seem to be more effective to select a small number of patients at clearly higher risk.

There is little literature on the incidence of SMM depletion determined directly at the C3 level, as the standard procedure is to calculate the SMMI at L3 from measurements made at C3 (the formula proposed by Swartz et al. [16]). However, because C3-to-L3 conversion formulas are complicated to use in routine practice, we evaluated the relationship with sarcopenia of the CSMA measured directly at C3. Jung et al. [21] have demonstrated, for patients with HNSCC, that direct CSMA measurement at C3 is a good predictor of overall survival; however, to our knowledge, no other studies evaluate PCF risk according to this determination of sarcopenia.

Our cut-off point was based on PCF formation. Considering the cut-off points most commonly used in the literature [7, 22], the proportion of our cohort presenting low SMM would be 80.2% according to Prado [22] (cut-offs of 52.4 cm^2^/m^2^ for men and 38.5 cm^2^/m^2^ for women), but only 22.1% according to Wendrich [7] (a cut-off of 43.2 cm^2^/m^2^). Those cut-off values vary greatly, because they were obtained on the basis of different dependent variables. The study by Prado et al. [22], carried out with a cohort of obese patients who had lung or gastrointestinal tract tumours, focused on determining a threshold that would define a significant association between decreased muscle mass and mortality in such patients, while the study by Wendrich et al. [7], performed in patients with HNSCC, aimed at obtaining a cut-off point related to the dose-limiting toxicity of chemotherapy.

### Low SMM as a PCF risk factor

Of the 86 patients evaluated in our study, 21 (24.4%) developed PCFs. The univariate analysis showed that low SMM was significantly associated with PCF formation (*p* = 0.011). Corroborating our results for a previous study [6], the other variables associated with PCF formation were tumour location and surgery type, specifically, hypopharyngeal tumours (*p* = 0.008) and extended TL with pharyngectomy (*p* = 0.002), respectively. Suture type was another variable associated with PCF formation in the univariate analysis, as manual suture implied a significant PCF risk. Note, however, that we consider this finding to be a consequence of selection bias, as only patients with endolaryngeal tumours treated with simple TL—with a lower a priori PCF risk [31]—were considered candidates for automatic suture closure.

**Fig. 1:**
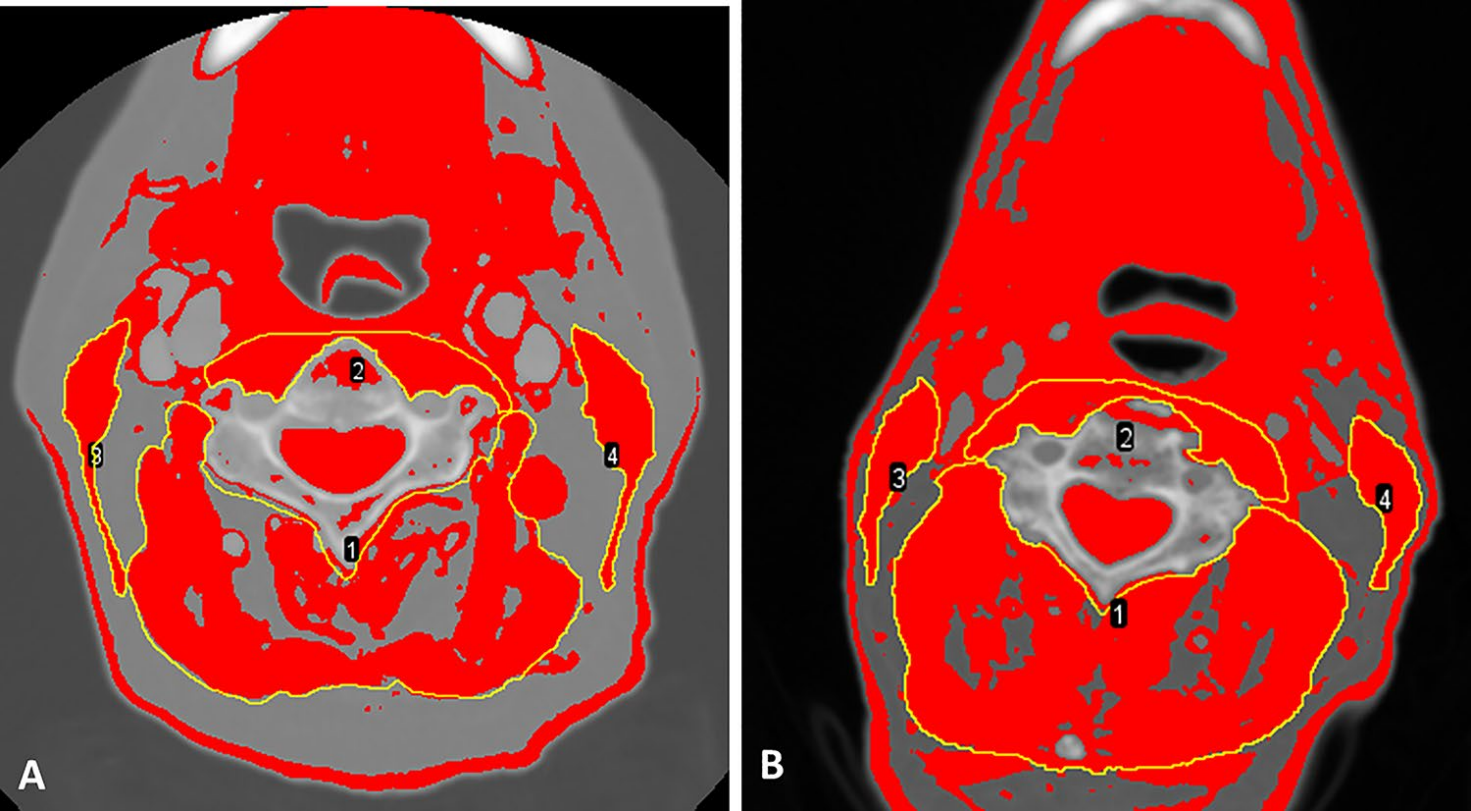
Images at the 3rd cervical vertebra corresponding to 2 patients with the same body mass index of 23 kg/m^2^. The red areas included in the manually designed yellow regions of interest corresponding to perivertebral musculature (labelled as 1 for nuchal and 2 for prevertebral muscles) and sternocleidomastoid muscles (labelled 3 and 4) are added to obtain the cross-sectional muscle area. The cross-sectional muscle areas of 29.4 cm^2^ in image A and 44 cm^2^ in image B would classify the patients as having low skeletal muscle mass and normal skeletal muscle mass, respectively

In line with the findings of other authors [9, 32–36], we found no significant relationship between BMI and PCF risk. However, for their multicentre study, Lansaat et al. [3] reported that the PCF rate was 2.7 times higher in underweight patients (BMI < 18 kg/m^2^) compared to normal weight patients, and Lebo et al. [1] similarly reported a higher PCF rate for underweight patients. Nonetheless, we agree with Prado et al. [22] argument that, since the BMI does not indicate the composition of each weight unit, the information it provides is incomplete. Exemplifying this argument are the C3 images for two patients in our study shown in Fig. 1: while both patients had the same BMI of 23 kg/m^2^, on the basis of their CSMA values of 29.4 cm^2^ (image A) and 43.9 cm2 (image B), they are classifiable as low SMM and normal SMM patients, respectively.

The multivariate analysis indicated that low SMM was the only variable that was significantly related to PCF risk, 9.88 times greater for patients with a CSMA of ≤ 35.5 cm^2^ than for patients with a CSMA of > 35.5 cm^2^ (*p* = 0.022). When CSMA was included in the multivariate analysis as an independent variable, the relationship that we identified in a previous study [6], namely, between the extent of surgery and PCF risk, no longer held; this finding would indicate that sarcopenia measured directly at C3 is, of the analysed variables, the most important predictor of PCF risk. It should be noted that the cut-off point used in the RPA was obtained specifically considering PCF formation as the dependent variable. The use of the same variable as a determinant cut-off to segregate the sample and as the dependent variable in the multivariate model is subject to bias. Therefore, studies need to be carried out to validate the paper of low SMM in patients treated with a laryngectomy.

Other authors have found SMM depletion to be associated with PCF formation in patients treated with TL [9, 10]. Achim et al. [9] analysed the CSMA at L3 for 70 patients treated with TL, finding that patients with sarcopenia developed 7.5 times more complications than patients without sarcopenia (95% CI 1.56–36.4), and also that PCF incidence was 32% greater in patients with low SMM (95% CI 1.13–1.53). For 235 patients treated with TL, Bril et al. [10] used the cut-off point proposed by Wendrich et al. [7] (43.2 cm^2^/m^2^) for CSMA at C3 converted to SMMI at L3, observing that SMM was reduced in 46.4% of cases, and also that patients with low SMM compared to patients with normal muscle mass more frequently developed PCFs (34.9% vs 20.6%; *p* = 0.02) and more frequently required surgical repair (*p* = 0.05).

Low SMM incidence as a risk factor for PCF was determined to be equivalent for our direct measurement of the CSMA at C3 and for the standard method of C3-to-L3 conversion and SMMI calculation. Our results would therefore suggest that measurement of the CSMA at C3 is a valid method for evaluating muscle mass, and therefore low SMM, in patients with HNSCC, while having the advantage of being a direct measurement that avoids the use of conversion formulas.

### Patients with low SMM

A relationship was observed between BMI and the CSMA at C3. Analysing percentages according to the WHO [27] BMI classification, we observed that low SMM was far more frequent (87.5%) in underweight patients (BMI < 18.5 kg/m^2^), and no low SMM cases (0.0%) were assessed in obese patients (BMI > 30 kg/m^2^). Corroborating our results, Cho et al. [37] reported a higher proportion of underweight patients in their depleted SMM group than in their normal SMM group (17% vs 4%; *p* < 0.001).

We found that the depleted SMM rate was higher in patients with extended surgeries. We attribute this finding to a higher tumour stage that requires complex surgeries including pharyngectomies with or without reconstruction. In turn, we believe that patients with advanced tumour stages present sarcopenia as a reflection of suboptimal nutritional status and tumour-related metabolic changes, such as preoperative anaemia or hypoalbuminemia. The increased rate of low SMM in patients sutured manually might be a consequence to the selection bias occurred, because automatic suture is only performed in patients with endolaryngeal tumours.

The main limitation of our study is that, given its retrospective nature, we were unable to evaluate the impact of certain variables that could influence PCF formation, such as weight loss, a history of tracheotomy [32], or perioperative transfusions [1, 4, 33, 38]. Another important limitation was the reduced number of patients and its heterogeneity in treatment strategy, which may lead to a selection bias. We were also unable to evaluate our patients’ muscle strength, a key aspect of sarcopenia diagnosis according to the EWGSOP [13].

Further studies need to be carried to validate the relationship between the CSMA at C3 and post-TL PCF formation, and to define cut-off points with a greater prognostic capacity.

## Conclusions

On the basis of a definition of low SMM associated with PCF formation, 20.9% of our cohort had preoperative SMM depletion as determined from the CSMA at C3. Our multivariate study of risk factors for PCF indicated low SMM to be the only independent risk factor: such patients had a near tenfold increased risk of PCF formation. The fact that measuring the CSMA at C3 obtained results similar to those obtained by calculating the SMMI at L3 would suggest that direct SMM assessment from C3 is a useful approach to evaluating post-TL PCF.

## Supplementary Information

Below is the link to the electronic supplementary material.

Supplementary file1 (DOCX 78 kb)

## Declarations

### Availability of data and materials

Data are available from the hospital software, SAP.
