# Supplementary material for: Low skeletal muscle mass assessed directly from the 3rd cervical vertebra can predict pharyngocutaneous fistula risk after total laryngectomy in the male population
Source: Eur Arch Otorhinolaryngol. 2021 Oct 19;279(2):853–63. doi: 10.1007/s00405-021-07127-3 (PMC8795024; doi:10.1007/s00405-021-07127-3)
Supplement: Supplementary file 1 — Supplementary file1 (DOCX 78 kb) [file 405_2021_7127_MOESM1_ESM.docx]

Supplementary Information

**Low skeletal muscle mass assessed directly from the 3rd cervical vertebra can predict pharyngocutaneous fistula risk after total laryngectomy in the male population**

European Archives of Oto-Rhino-Laryngology and Head and Neck

Maria Casasayas,^a^ Jacinto García-Lorenzo, Beatriz Gómez-Ansón, Victoria Medina, Alejandro Fernández, Miquel Quer, Xavier León

^a^ Servicio de Otorrinolaringología. Hospital de la Santa Creu i Sant Pau, Universitat Autònoma de Barcelona, Barcelona, Spain

E-mail: [mcasasayas@santpau.cat](mailto:mcasasayas@santpau.cat)

**Fig. S1** Dot plots showing correlation between measurements of the cross-sectional muscle area (CSMA) at the 3rd cervical vertebra (C3) for 10 computed tomography images. A. Obtained by the same researcher on 2 different occasions (intraobserver reproducibility). B. Obtained by 2 different researchers (interobserver reproducibility). All measurements are expressed in cm2


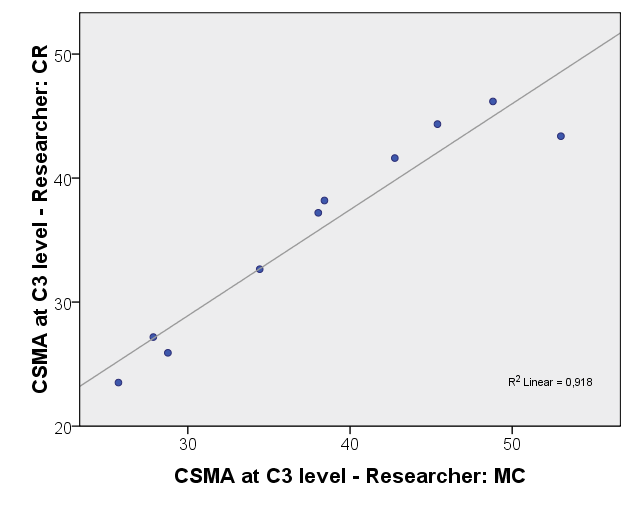

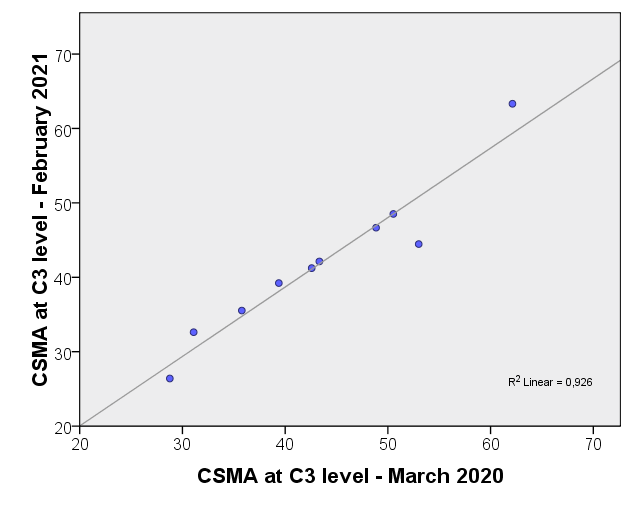


*P=0.0001*

*P=0.0001*


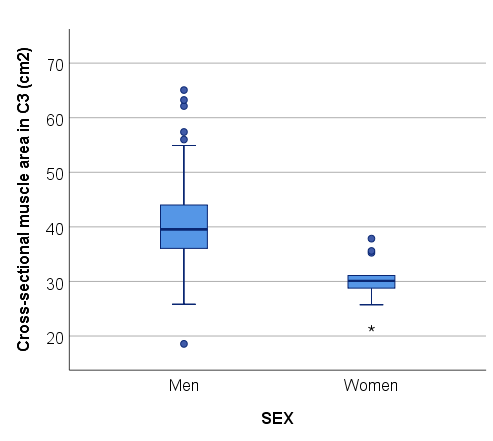
**Fig. S2** Box plot showing cross sectional muscle area (cm2) mean and distribution according to sex. Mean area for men was 41.02cm2 (SD 7.9cm2), while mean area for women was 30.22cm2 (SD 4.3cm2) (p=0.000)

**B**

**A**

**Table S1.** Pharyngocutaneous fistula risk: multivariate analysis including the skeletal muscle mass index estimated at the 3rd lumbar vertebra.

| **Characteristic** | |  | **PCF HR (CI 95%)** | **p-value** |
| --- | --- | --- | --- | --- |
| **Age** | (Continuous variable) | | 0.95 (0.87-1.03) | 0.218 |
| **Tobacco/alcohol use** | Moderate | | 1 | 0.051 |
|  | Heavy | | 0.15 (0.02-1.01) |  |
| **ASA physical status** | I-II | | 1 | 0.838 |
|  | III-IV | | 0.86 (0.20-3.73) |  |
| **Diabetes mellitus** | No | | 1 | 0.169 |
|  | Yes | | 3.04 (0.62-14.90) |  |
| **Body mass index, kg/m2** | (Continuous variable) | | 1.13 (0.94-1.36) | 0.182 |
| **SMMI at L3**  (Low SMM = ≤47.7 cm2/m2) | >47.7cm2/m2 | | 1 | 0.031* |
|  | ≤47.7 cm2/m2 | | 8.50 (1.22-47.04) |  |
| **Subsite location** | Supraglottis | | 1 |  |
|  | Glottis | | 1.558 (0.25-9.80) | 0.625 |
|  | Hypopharynx | | 1.29 (0.11-14.86) | 0.839 |
| **Tumour stage (pT)** | pT2 | | 1 |  |
|  | pT3 | | 1.13 (0.11-11.87) | 0.919 |
|  | pT4 | | 1.22 (0.16-9.28) | 0.846 |
| **Previous radiotherapy** | No | | 1 | 0.594 |
|  | Yes | | 0.67 (0.15-2.97) |  |
| **Surgery type** | Simple TL | | 1 | 0.096 |
|  | Extended TL +/- reconstruction | | 7.93 (0.69-90.95) |  |
| **Automatic suture** | No | | 1 | 0.195 |
|  | Yes | | 0.31 (0.05-1.84) |  |
| **Neck dissection** | No | | 1 |  |
|  | Unilateral | | 0.96 (0.08-11.73) | 0.972 |
|  | Bilateral | | 1.08 (0.15-8.02) | 0.938 |
| **Voice prosthesis** | No | | 1 | 0.497 |
|  | Yes | | 0.38 (0.02-6.20) |  |
| **Preoperative anaemia** | No | | 1 | 0.831 |
|  | Yes | | 0.84 (0.17-4.09) |  |
| **Postoperative anaemia** | No | | 1 | 0.424 |
|  | Yes | | 3.05 (0.20-47.04) |  |

*Statistical significance p<0.05. ASA, American Society of Anesthesiologists; HR, hazard ratio; L3, 3rd lumbar vertebra; PCF, pharyngocutaneous fistula; SMMI, skeletal muscle mass index; TL, total laryngectomy.

**Table S2.** Cross-sectional muscle area at the 3rd cervical vertebra according to different variables.

| **Characteristic** |  | | **Mean (SD) CSMA**  **at C3, cm^2^** | | **p-value** |
| --- | --- | --- | --- | --- | --- |
| **Age, years** | | Pearson correlation | | -0.138 | 0.207 |
| **Tobacco/alcohol use** | | Moderate | | 39.66 (3.7) | 0.249 |
|  |  | Heavy | | 41.28 (8.4) |  |
| **ASA physical status** | | I-II | | 40.78 (6.8) | 0.809 |
|  |  | III-IV | | 41.20 (8.8) |  |
| **Diabetes mellitus** | | No | | 39.95 (7.3) | 0.047* |
|  |  | Yes | | 43.70 (8.8) |  |
| **Height (m)** | | Pearson correlation | | 0.130 | 0.233 |
| **Weight (kg)** | | Pearson correlation | | 0.480 | 0.0001* |
| **Body mass index, kg/m2** | | Pearson correlation | | 0.469 | 0.0001* |
| **Subsite location** | | Supraglottis | | 39.22 (8.2) | 0.043* |
|  |  | Glottis | | 42.67 (8.0) |  |
|  |  | Hypopharynx | | 37.12 (3.7) |  |
| **Tumour stage (pT)** | | pT2 | | 42.00 (7.3) | 0.422 |
|  |  | pT3 | | 42.76 (10.4) |  |
|  |  | pT4 | | 40.17 (7.0) |  |
| **Previous radiotherapy** | | No | | 39.95 (7.8) | 0.219 |
|  |  | Yes | | 42.05 (7.9) |  |
| **Surgery type** | | Simple TL | | 42.02 (8.4) | 0.001* |
|  |  | Extended TL +/- reconstruction | | 37.38 (3.6) |  |
| **Automatic suture** | | No | | 40.13 (8.6) | 0.166 |
|  |  | Yes | | 42.61 (6.0) |  |
| **Neck dissection** | | No | | 43.16 (9.3) | 0.243 |
|  |  | Unilateral | | 43.44 (9.4) |  |
|  |  | Bilateral | | 40.14 (7.2) |  |
| **Voice prosthesis** | | No | | 40.56 (7.9) | 0.158 |
|  |  | Yes | | 44.32 (6.9) |  |
| **Preoperative protein, g/L †** | | Pearson correlation | | -0.181 | 0.240 |
| **Preoperative albumin, g/L ‡** | | Pearson correlation | | 0.093 | 0.537 |
| **Preoperative haemoglobin, g/L** | | Pearson correlation | | -0.007 | 0.948 |
| **Postoperative haemoglobin, g/L** | | Pearson correlation | | -0.010 | 0.929 |

*Statistical significance p<0.05. † Data available from 44 patients. ‡ Data available from 46 patients.

ASA, American Society of Anesthesiologists; C3, 3rd cervical vertebra; CSMA, cross-sectional muscle area; PCF, pharyngocutaneous fistula; TL, total laryngectomy.
